# Vitamin D and suicidality: a Chinese early adolescent cohort and Mendelian randomization study

**DOI:** 10.1017/S2045796023000665

**Published:** 2023-08-09

**Authors:** Mengyuan Yuan, Yonghan Li, Junjie Chang, Xueying Zhang, Shaojie Wang, Leilei Cao, Yuan Li, Gengfu Wang, Puyu Su

**Affiliations:** 1 Department of Maternal, Child and Adolescent Health, School of Public Health, Anhui Medical University, Hefei, Anhui, China; 2 Key Laboratory of Population Health Across Life Cycle (Anhui Medical University), Ministry of Education of the People’s Republic of China, Hefei, Anhui, China; 3 Department of Environmental Medicine and Public Health, Icahn School of Medicine at Mount Sinai, New York, NY, USA; 4 Anhui Provincial Key Laboratory of Population Health and Aristogenics, Hefei, Anhui, China

**Keywords:** cohort, early adolescents, Mendelian randomization, suicidality, vitamin D

## Abstract

**Aims:**

Previous cross-sectional and case–control studies have proposed that decreased vitamin D levels are positively correlated with the risk of suicidality in adults. However, limited studies have examined the association between vitamin D and suicidality in adolescents. This study aimed to investigate the relationship between serum vitamin D and suicidality risk among early adolescents.

**Methods:**

Data were obtained from a Chinese early adolescent cohort. In this cohort, seventh-grade students from a middle school in Anhui Province were invited to voluntarily participate in the baseline assessments and provide peripheral blood samples (in September 2019). The participants were followed up annually (in September 2020 and September 2021). Serum 25-hydroxyvitamin D [25(OH)D] and vitamin D–related single-nucleotide polymorphisms at baseline were measured in November 2021. Traditional observational and Mendelian randomization (MR) analyses were performed to examine the relationship between serum 25(OH)D at baseline and the risk of baseline and incident suicidality (i.e., suicidal ideation [SI], plans and attempts).

**Results:**

Traditional observational analysis did not reveal a significant linear or non-linear association of serum 25(OH)D concentration with the risks of baseline and 2-year incident suicidality in the total sample (*P* > .05 for all). Sex-stratified analysis revealed a non-linear association between the 25(OH)D concentration and the risk of baseline SI in women (*P*_overall_ = .002; *P*_non-linear_ = .001). Moreover, the risk of baseline SI in the 25(OH) insufficiency group was lower than that in the 25(OH) deficiency group in the total sample (odds ratio [OR] = 0.69, 95% confidence interval [CI] = 0.51–0.92, *P* = .012). This difference remained significant in women (OR = 0.59, 95% CI = 0.40–0.87, *P =* .008) but not in men (OR = 0.78, 95% CI = 0.53–1.15, *P =* .205). Additionally, both linear and non-linear MR analyses did not support the causal effect of serum 25(OH)D concentration on the risk of baseline, 1-year and 2-year incident suicidality (*P* > .05 for all).

**Conclusions:**

This study could not confirm the causal effect of vitamin D on suicidality risk among Chinese early adolescents. Future studies must confirm these findings with a large sample size.

## Introduction

Over the past few decades, adolescent suicide has become a major public health concern. According to the United Nations International Children’s Emergency Fund, suicide is the fifth leading cause of death in adolescents aged 10–19 years worldwide (UNICEF, 2021). Suicidality, including suicidal ideation (SI), plans (SP) and attempts (SA), is reported to be the precursor of suicide death (Nock *et al.*, 2008). The global prevalence rates of SI, SP and SA among adolescents aged 12–15 years are estimated to be 16.5%, 16.5% and 16.4%, respectively (Tang *et al.*, 2020). In China, a cross-sectional study involving 7,986 middle school students reported that the prevalence rates of SI, SP and SA were 22.9%, 10.8% and 4.7%, respectively (Xie *et al.*, 2022). As the prevalence of suicidality is high in early adolescents, there is an urgent need to identify modifiable risk factors to inform the development of effective suicide prevention and intervention strategies.

Recently, the potential beneficial roles of vitamin D in mental and brain health have increasingly gained attention (Focker *et al.*, 2017). Previous observational studies and interventional trials have investigated the association between vitamin D status or supplementation and several common psychiatric traits (Cheng *et al.*, 2020; Focker *et al.*, 2017; Glabska *et al.*, 2021; Guzek *et al.*, 2021; Jamilian *et al.*, 2019), including depression (Li *et al.*, 2019; Ronaldson *et al.*, 2022), anxiety (de Koning *et al.*, 2017; Maddock *et al.*, 2013) and psychosis (Lally *et al.*, 2019). To the best of our knowledge, limited epidemiological studies have investigated the association between vitamin D and suicidality. Additionally, most of these epidemiological studies were based on cross-sectional or case–control designs and reported inconsistent results (supplementary eTable 1). Some studies reported that peripheral vitamin D was significantly associated with the risks of SI, SA and completed suicide (Grudet *et al.*, 2014; Kim *et al.*, 2020; Umhau *et al.*, 2013). However, other studies have reported that vitamin D was not significantly associated with SI (Park *et al.*, 2016). One possible explanation for these discrepant results is the usage of different cut-off values for vitamin D levels in these studies [e.g. >30 ng/mL for sufficiency (Park *et al.*, 2016); ≥20 ng/mL for sufficiency (Kim *et al.*, 2020); see details in eTable 1]. Other factors, such as differences in adjusted covariates and population characteristics, may also have contributed to the discrepant results (see eTable 1). Recent studies have suggested the threshold of vitamin D concentration below which disease risk and vitamin D supplementation benefits can be observed, indicating a non-linear relationship between vitamin D and some chronic diseases (Scragg, 2018). Therefore, both linearity and non-linearity should be considered when investigating the relationship between vitamin D and suicidality risk.

Traditional observational studies are susceptible to confounding bias and reverse causality, limiting their ability to draw causal inferences. Mendelian randomization (MR), as a natural analogue of randomized controlled trials, offers an opportunity to detect possible causal effects in a time-efficient and cost-effective manner and has been applied in the field of nutritional psychiatry (Carnegie *et al.*, 2020; Wootton *et al.*, 2022). Recently, the causal role of vitamin D in some psychiatric conditions, such as depression (Libuda *et al.*, 2019; Mulugeta *et al.*, 2020) and schizophrenia (Taylor *et al.*, 2016), has been examined using MR. However, traditional MR is based on the hypothesis that the association between exposure and outcome is linear. To overcome this limitation, non-linear MR has been developed and used to investigate the causal effect of vitamin D on some medical outcomes (Navale *et al.*, 2022; Sutherland *et al.*, 2022; Zhou *et al.*, 2022). Thus, both linear and non-linear MR methods should be employed to explore the causal effect of vitamin D on suicidality.

One case–control study constructed a genetic risk score (GRS) using four vitamin D–related gene polymorphisms (namely, DHCR7-rs12785878, CYP2R1-rs10741657, GC-rs2282679 and CYP24A1-rs6013897). GRS analysis showed a higher risk of attempted suicide with a greater number of low vitamin D alleles in men but not in women (Wei *et al.*, 2021), indicating a sex-specific relationship. Additionally, the relationship between vitamin D and other mental problems was reported to be sex-specific (Hinata *et al.*, 2023; Wang *et al.*, 2022). However, most previous studies on vitamin D and suicidality matched sex or adjusted sex as a covariate in the statistical models, which would overlook the sex differences. Therefore, sex-stratified analysis is needed to examine whether vitamin D affects suicidality in a sex-dependent manner.

Additionally, previous studies examining the relationship between vitamin D and suicidality were mainly conducted in adults. To the best of our knowledge, only one case–control study was conducted in adolescents and revealed that the mean vitamin D levels were significantly decreased in patients who attempted suicide (Gokalp, 2020). Early adolescence (10–14 years of age), which represents a transition period from childhood to adolescence, is characterized by immense cognitive and emotional changes. Previous studies have reported that the incidence of the first onset of suicidality peaks during early adolescence (van Vuuren *et al.*, 2021) and increases with age. Meanwhile, the prevalence of vitamin D deficiency tends to increase rapidly from childhood to early adolescence (Hu *et al.*, 2022a) due to increased academic burden and decreased outdoor activities. Therefore, there is a need to investigate the causal effect of vitamin D on suicidality in early adolescence, which will provide new insights for implementing a feasible programme for suicide prevention and intervention.

This study aimed to use data from a Chinese early adolescent cohort to investigate the relationship of serum 25-hydroxyvitamin D [25(OH)D, an improved indicator of vitamin D stores] with the risk of suicidality. Traditional statistical methods were used to investigate the observational association of the baseline 25(OH)D concentration or levels with baseline and incident suicidality. Next, linear and non-linear MR methods were employed to establish the causal association of baseline 25(OH)D concentration with baseline and incident suicidality. Additionally, sex-stratified analysis was performed to determine sex differences in the observational and causal associations of baseline 25(OH)D with baseline and incident suicidality risk.

## Methods

This study was approved by the ethical committee of Anhui Medical University (No. 20180083) and complied with the guidelines of the Declaration of Helsinki. All school leaders, adolescents and their caregivers participating in the study signed an informed consent form. The Strengthening the Reporting of Observational studies in Epidemiology (STROBE) and STROBE-MR guidelines were followed for reporting observational and MR studies, respectively (Skrivankova *et al.*, 2021; von Elm *et al.*, 2007). Detailed information on these two checklists is shown in eTables 2 and 3.

### Study settings and participants

In this study, data were obtained from the Chinese Early Adolescents Cohort (CEAC) (Wang *et al.*, 2022). The CEAC is an annual three-wave longitudinal study to examine the risk factors associated with emotional and behavioural problems in Chinese early adolescents. This study included 1,814 participants from one middle school in Huaibei City (Anhui Province, China) who provided written informed consent. The exclusion criteria were as follows: severe liver/renal diseases, physical disabilities, severe injuries or infection, the use of psychiatric medications and incomplete questionnaire data on covariates and/or suicidality. In total, 1,426 students with complete data on serum 25(OH)D concentration, covariates and suicidality were included for baseline suicidality analysis using traditional observational methods. Of these 1,426 participants, only those students who reported no suicidality at baseline were included for incident suicidality analysis using the traditional observational method (SI: 1,083; SP: 1,282; SA: 1,404). In total, 1,411 students with complete data on serum 25(OH)D concentration, covariates, suicidality and vitamin D–related genotypes were included for baseline suicidality analysis using the MR method. Of these 1,411 participants, only those students who reported no suicidality at baseline were included for incident suicidality analysis using the MR method (SI: 1072; SP: 1,269; SA: 1,389). Further details on the selection process of the study participants are provided in online supplementary eFigure 1.

### Measurements

#### Baseline serum 25(OH)D concentration

At baseline, fasting blood samples were collected from participants in the morning. The blood samples were centrifuged to obtain the serum. The serum samples were aliquoted and stored at −80℃ until further use. In December 2021, the baseline serum 25(OH)D concentration (ng/mL) was measured using the LIASON 25-OH vitamin D TOTAL assay (DiaSorin, Inc.). Based on previous studies (Cheng *et al.*, 2022; Hu *et al.*, 2022b), the serum 25(OH)D levels were grouped into the following three categories: sufficiency (≥30 ng/mL), insufficiency (≥20 and < 30 ng/mL) and deficiency (<20 ng/mL). The distribution of serum 25(OH)D concentration in blood samples was approximately normal (eFigure 2).

**Figure 1.**
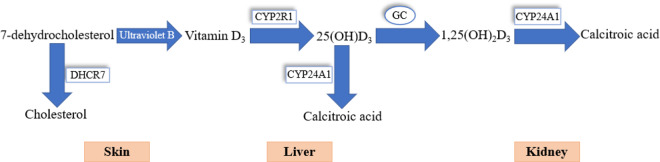
The role of proteins encoded by the selected candidate genes in the vitamin D synthesis and metabolism. Note. *DHCR7* and *CYP2R1* are involved in vitamin D synthesis. *GC, CYP24A1* and *CYP27B1* are involved in vitamin D metabolism.

#### Baseline and incident suicidality

At wave I, suicidality in the past 6 months was assessed. However, at waves II and III, suicidality in the past year was assessed. In each wave, the following three questions were asked: (1) have you ever thought about killing yourself? (SI), (2) have you ever planned to end your life? (SP), and (3) have you ever attempted to take your own life? (SA). The responses were recorded as yes or no. Therefore, SI, SP and SA were treated as dichotomous variables. For baseline suicidality analysis, participants who reported having suicidality at wave I were regarded as having baseline suicidality. For incident suicidality analysis, we calculated two incident suicidality outcomes: (1) 1-year incident suicidality (i.e. reported no suicidality at wave I but reported having suicidality at wave II) and (2) 2-year incident suicidality (i.e. reported no suicidality at wave I but reported having suicidality at waves II and/or III).

#### Calculation of vitamin D polygenic score

DNA was extracted from the peripheral blood samples collected at baseline. Based on the Chinese genetic background, single-nucleotide polymorphisms (SNPs) were mainly selected in the following two steps: selection of common vitamin D metabolism-related genes (Cheung *et al.*, 2013; Lu *et al.*, 2012; Robien *et al.*, 2013; Zhang *et al.*, 2012; Zhou *et al.*, 2014) (Fig. 1) and selection of key SNPs in these genes (Ahn *et al.*, 2010; Wang *et al.*, 2010) Finally, five SNPs (rs10741657, rs6013897, rs1790349, rs4588 and rs7041) located in four genes were selected (Table 1). In December 2021, SNPs were genotyped using the Sequenom MassARRAY iPLEX platform (Agena Bioscience, Inc., San Diego, CA, USA). An unweighted vitamin D polygenic score (VD-PGS) was calculated for further MR analysis (Supplementary eMethods). The higher the VD-PGS, the lower the 25(OH)D concentration (eTable 4).

**Table 1.** Basic characteristics of the candidate genes

Gene	Location	Function in the vitamin D pathway	Selected SNPs
*CYP2R1*	11p15.2	Encoding vitamin D 25-hydroxylase	rs10741657
*CYP24A1*	20q13	Encoding vitamin D_3_ 25-hydroxylase	rs6013897
*DHCR7*	11q13.4	Encoding 7-dehydrocholesterol reductase	rs1790349
*GC*	4q12-q13	Encoding vitamin D binding protein	rs4588^a^ rs7041

SNPs, single-nucleotide polymorphism

a indicates that rs4588 is in high-LD with rs2282679.

#### Identification of genetic instrument variables

The selection of instrument variables (IVs) needs to satisfy three IV assumptions (Lousdal, 2018). To reduce weak genetic instrument bias, the association between each SNP and vitamin D was examined. Two SNPs (rs6013897 and rs1790349) that were not significantly related to serum 25(OH)D concentration were excluded (eTable 5). Finally, three SNPs (rs10741657, rs4588 and rs7041) were included (Supplementary eMethods). To assess the adequacy of IVs, the serum 25(OH)D concentration was regressed on VD-PGS with adjustment for age and sex to obtain the proportion of variance explained by the specified SNPs (i.e. *R*^2^). The *F*-statistic was calculated as follows: *F* =
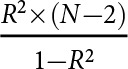
 (*N* represents the total sample size) (Yin *et al.*, 2022). Generally, *F* > 10 was considered to have sufficient genetic instrument strength (Pierce *et al.*, 2011). Additionally, the association between VD-PGS and potential risk factors for suicidality was examined to explore the presence of horizontal pleiotropic effects.

#### Covariates

Potential covariates were identified based on previous literature investigating the causal risk factors for vitamin D among children and adolescents (Arguelles *et al.*, 2009; Hu *et al.*, 2017; Mulugeta *et al.*, 2020; Revez *et al.*, 2020; Tolppanen *et al.*, 2012). The predictors of adolescent suicidality (Abreu *et al.*, 2018; Carballo *et al.*, 2020; Miranda-Mendizabal *et al.*, 2019), especially in China (Liu *et al.*, 2019), were identified. Next, a directed acyclic graph was used to identify a set of minimal sufficient covariates (eFigure 3). Mood disorders, anxiety disorders and substance misuse are the most prevalent psychiatric morbidities in individuals with suicidality (Knipe *et al.*, 2019). Among Chinese early adolescents, attention-deficit/hyperactivity disorder (ADHD), oppositional defiant disorder (ODD), generalized anxiety disorder and major depressive disorder are the most common psychiatric disorders (Shen *et al.*, 2018). The trait-impulsivity etiological model posits impulsivity as the underlying construct shared in many externalizing problems (e.g. substance misuse, ADHD and ODD) (Beauchaine *et al.*, 2017; Martel *et al.*, 2017; Rodenacker *et al.*, 2018), which could strongly predict adolescent suicidality (Gorlyn, 2005). Thus, depression, anxiety and impulsivity were selected as comorbid psychiatric covariates in this study. Information on the prevalence of psychiatric comorbidities in suicidality among the study participants is shown in eTable 6. Finally, adjusted covariates in the traditional observational analysis of this study included sex, age, household registration, family economy, paternal education, maternal education, moderate physical activity, vigorous physical activity and psychiatric comorbidities (i.e. impulsivity, depression and anxiety). Furthermore, only sex and age were adjusted in MR analysis. Detailed information on these covariates is provided in eTable 7.

### Statistical analysis

Descriptive statistics including the mean [standard deviation (SD)] for continuous variables and the number of participants (%) for categorical variables were used to describe the basic characteristics of the study participants.

For traditional observational analysis, logistic regression models were constructed to calculate odds ratios (ORs) for suicidality based on the continuous serum 25(OH)D concentration and categorical 25(OH)D levels with adjustments for potential covariates. Additionally, fractional polynomial models were constructed to determine the appropriate functional form of continuous 25(OH)D concentration. Likelihood ratio tests were performed to compare the best-fitting fractional polynomial model against the linear model. A *P*-value <.05 indicated the presence of a non-linear association.

For MR analysis, both linear [the ratio of coefficients method (Burgess *et al.*, 2017)] and non-linear MR (Staley and Burgess, 2017) methods were employed to examine the association between genetically determined serum 25(OH)D concentration and the risk of suicidality using individual-level data. For linear MR, linear regression models were employed to estimate the effect of the genetic instrument (VD-PGS) on vitamin D (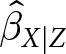) and logistic regression models were subsequently used to estimate the effect of VD-PRS on suicidality risk (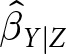). Both regressions were adjusted for age and sex. Linear MR estimates can be expressed as 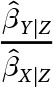, and their 95% confidence intervals (CIs) can be computed using a first-order Taylor series approximation (Arvanitis *et al.*, 2021). For non-linear MR, fractional polynomial models were fitted to localized average causal effects (LACEs) using a meta-regression method. Briefly, the study sample was split into 10 quantiles according to the instrument-free 25(OH)D (residuals of serum 25(OH)D after regressing on VD-PGS). Within each quantile, a linear MR estimate was fitted, known as LACEs. Next, meta-regression of LACEs was performed against quantile-specific mean 25(OH)D by fitting a range of fractional polynomial models of Degrees 1 and 2. The best-fitting model was selected. The following three statistical non-linear tests were reported: the fractional polynomial test, which compares the best-fitting fractional polynomial model of Degree 1 against the linear model; the quadratic test, which assesses the linear trend among LACEs; and the Cochran’s *Q*, which assesses differences in LACEs across 10 quantiles. Two heterogeneity tests (Cochran’s *Q* and trend tests) were performed to examine whether the association between VD-PGS and 25(OH)D was constant across 10 quantiles of vitamin D concentration. The statistical power for MR analysis was calculated using the online mRnd website (https://shiny.cnsgenomics.com/mRnd/) (Brion *et al.*, 2013). Notably, bidirectional MR was not performed due to the unavailability of genotype data on suicidality-related SNPs in this study.

In the sex-stratified analysis, male and female samples were separately analyzed to determine sex differences in the relationship between serum 25(OH)D and suicidality risk. All statistical analyses and figures were generated using R statistical software (version 4.1.0). A two-sided *P*-value <.05 was considered statistically significant.

## Results

A total of 1,426 students (60.8% of males; mean [SD] = 12.48 [0.48]) were included for traditional observational analysis. The mean serum 25(OH)D concentration was 23.12 ng/mL (SD = 5.76 ng/mL). Approximately 30.7%, 57.0% and 12.3% of the participants exhibited 25(OH)D deficiency, insufficiency and sufficiency, respectively. The baseline characteristics of the study participants are presented in eTable 8. At baseline, 24.1%, 10.1% and 1.5% of the participants reported SI, SP and SA, respectively (Table 2). Among the participants who did not report suicidality at baseline, 12.8%, 7.8% and 2.8% reported 1-year incident SI, SP and SA, respectively (Table 2). A total of 25.6%, 13.4% and 4.1% of participants who had no baseline suicidality reported 2-year incident SI, SP and SA, respectively (Table 2). Additionally, 1,411 participants included in the traditional observational analysis who had data on vitamin D–related SNPs were included for subsequent MR analysis. The characteristics of the participants included in the MR analysis were similar to those included in the traditional observational analysis (eTable 8).

**Table 2.** Traditional observational associations between baseline serum 25(OH)D concentration and the risk of suicidality in the total sample

		Logistic regression models		Fractional polynomial models	
Outcomes	*N* (%)	OR (95% CI)	*P*_linear_	*P*_overall_	*P*_non-linear_
Baseline suicidality					
Suicidal ideation	343 (24.1)	1.00 (0.97, 1.02)	.691	.212	.128
Suicidal plans	144 (10.1)	0.98 (0.95, 1.02)	.293	.604	.636
Suicidal attempts	22 (1.5)	0.97 (0.89, 1.06)	.496	.642	.604
One-year incident suicidality					
Suicidal ideation	182 (12.8)	1.03 (1.00, 1.06)	.036*	.029*	.392
Suicidal plans	111 (7.8)	1.03 (1.00, 1.07)	.058	.289	.887
Suicidal attempts	40 (2.8)	1.01 (0.95, 1.07)	.855	.563	.304
Two-year incident suicidality					
Suicidal ideation	277 (25.6)	1.01 (0.99, 1.04)	.368	.367	.320
Suicidal plans	172 (13.4)	1.02 (0.99, 1.04)	.289	.727	.790
Suicidal attempts	58 (4.1)	1.00 (0.95, 1.05)	.986	.754	.595

Note. All models adjusted for age, sex, household registration, family economy, paternal education, maternal education, moderate physical activity, vigorous physical activity, impulsivity, depression and anxiety. *P*_linear_ indicates *P-*value for logistic regression model. *P*_overall_ indicates *P-*value for comparing the best-fitting second-degree model against the null model. *P*_non-linear_ indicates *P*-value for comparing the best-fitting second-degree model against the linear model.

* indicates *P*-value <.05.

### Instrument validation

After adjusting for sex and age, VD-PGS was associated with the serum 25(OH)D concentration (β = 0.199, standardized error = 0.026, *P* < .001), explaining 3.97% (*R*^2^) of the variation (*F*-statistic = 58.25). No significant heterogeneity was observed in the association between VD-PGS and serum 25(OH)D concentration across 10 quantiles of residual serum 25(OH)D concentration for any suicidality outcomes (*P* > .05 for all; eFigure 4). Thus, the association between VD-PGS and 25(OH)D associations in the 1st and 10th quantiles appeared to be outliers (eFigure 4). VD-PGS was not associated with potential confounders (*P* = .204–.977; eTable 9).

### Traditional observational analysis

In the multivariable-adjusted logistic regression models (Table 2), the serum 25(OH)D concentration was not significantly associated with the risk of baseline (OR = 0.97–1.00; *P*_linear_ > .05 for all) and 2-year incident (OR = 1.00–1.02; *P*_linear_ > .05 for all) suicidality. However, the 25(OH)D concentration was significantly associated with the risk of 1-year incident SI (OR [95% CI] = 1.03 [1.00–1.06], *P*_linear_ = .036). Additionally, the serum 25(OH)D concentration did not exhibit a significant non-linear association with the risk of baseline (*P*_non-linear_ = .128–.636) and incident (*P*_non-linear_ = .304–.887 for 1-year incidence; *P*_non-linear_ = .320–.790 for 2-year incidence) suicidality outcomes (Table 2). Compared with those in the 25(OH)D deficiency group, the risk of baseline SI was significantly lower (OR [95% CI], 0.69 [0.51–0.92]; *P* = .012) and the risks of incident SIs were higher (OR [95% CI] = 1.63 [1.11–2.41], *P* = .013 for 1-year incident SI; OR [95% CI] = 1.42 [1.02–1.96], *P* = .037 for 2-year incident SI) in the 25(OH)D insufficiency group (Table 3). The risk of baseline or incident suicidality outcomes was not significantly different between the 25(OH)D deficiency and sufficiency groups (Table 3).

**Table 3.** Traditional observational associations between baseline serum 25(OH)D levels and the risk of suicidality in the total sample

	Baseline 25(OH)D levels			
	Insufficiency		Sufficiency	
Outcomes	OR (95% CI)	*P*-value	OR (95% CI)	*P*-value
Baseline suicidality				
Suicidal ideation	0.69 (0.51, 0.92)	.012*	0.99 (0.64, 1.54)	.971
Suicidal plans	0.74 (0.50, 1.09)	.129	0.57 (0.28, 1.16)	.123
Suicidal attempts	0.87 (0.34, 2.17)	.758	0.41 (0.05, 3.45)	.409
One-year incident suicidality				
Suicidal ideation	1.63 (1.11, 2.41)	.013*	1.04 (0.55, 1.95)	.914
Suicidal plans	1.41 (0.89, 2.25)	.145	1.21 (0.59, 2.48)	.608
Suicidal attempts	0.99 (0.48, 2.01)	.973	0.79 (0.21, 2.93)	.728
Two-year incident suicidality				
Suicidal ideation	1.42 (1.02, 1.96)	.037*	1.06 (0.64, 1.77)	.809
Suicidal plans	1.44 (0.99, 2.12)	.059	1.05 (0.57, 1.94)	.864
Suicidal attempts	0.89 (0.50, 1.61)	.712	0.67 (0.22, 2.07)	.489

OR, odds ratio; CI, confidence interval.

All logistic regression models were adjusted for sex, age, household registration, family economy, paternal education, maternal education, moderate physical activity, vigorous physical activity, impulsivity, depression and anxiety, with reference to 25(OH)D deficiency group.

* indicates *P*-value <.05.

### MR analysis

Linear MR analysis revealed that no significant linear relationship between serum 25(OH)D concentration and the risk of baseline, 1-year and 2-year incident suicidality outcomes (*P*_linear_ > .05 for all; Table 4). Additionally, non-linear MR analysis revealed no significant non-linear association between 25(OH)D concentration and the risk of baseline suicidality (*P-*values for the fractional polynomial, Cochran’s *Q* and quadratic tests were in the range of .658–1.000, 139–.834 and .754–.975, respectively). There was no significant non-linear MR association between 25(OH)D concentration and the risk of 1-year (*P-*values for the fractional polynomial, Cochran’s *Q* and quadratic tests were in the range of .622–.739, .392–.695 and .543–.615, respectively) or 2-year (*P-*values for the fractional polynomial, Cochran’s *Q* and quadratic tests were in the range of .343–.530, .403–.594 and .091–.331, respectively) incident suicidality (Table 4). Detailed information on the power calculation for MR analysis is provided in supplementary eTable 10.

**Table 4.** MR associations between serum 25(OH)D concentration and the risk of suicidality in the total sample

	Linear MR		Non-linear MR		
Outcomes	OR (95% CI)	*P*_linear_	*P*_fp_	*P*_quadratic_	*P*_Cochrane’s Q_
Baseline suicidality					
Suicidal ideation	0.95 (0.85, 1.05)	.310	1.000	.975	.535
Suicidal plans	0.96 (0.82, 1.11)	.558	.658	.754	.139
Suicidal attempts	0.93 (0.64, 1.35)	.696	.907	.907	.834
One-year incident suicidality					
Suicidal ideation	1.05 (0.91, 1.21)	.480	.622	.392	.543
Suicidal plans	1.01 (0.85, 1.20)	.900	.739	.695	.564
Suicidal attempts	0.95 (0.72, 1.26)	.744	.712	.445	.615
Two-year incident suicidality					
Suicidal ideation	1.11 (0.99, 1.26)	.084	.343	.091	.403
Suicidal plans	1.06 (0.92, 1.22)	.423	.530	.331	.594
Suicidal attempts	1.00 (0.80, 1.27)	.976	.489	.216	.548

OR, odds ratio; CI, confidence interval; MR, Mendelian randomization.

All MR analyses adjusted for sex and age. *P*_linear_ indicates *P-*value for logistic regression model. *P*_fp_ indicates *P*-value for fractional polynomial test. *P*_quadratic_ indicates *P*-value for quadratic test. *P*_Cochrane’s *Q*_ indicates *P*-value for Cochran’s *Q* test.

### Sex differences

Sex-stratified traditional observational analysis revealed no linear or non-linear relationship between serum 25(OH)D concentration and the risk of baseline or incident suicidality outcomes among men (all *P*_linear_ and *P*_non-linear_ > .05; eTable 11). However, the 25(OH)D concentration exhibited a significant non-linear relationship with the risk of baseline SI in women (*P*_non-linear_ = .001; eTable 12; eFigure 5). When the 25(OH)D concentration was categorized into three levels, the risk of baseline or incident suicidality in the 25(OH)D insufficiency/sufficiency group was not significantly different from that in the 25(OH)D deficiency group among men (all *P* > .05; eTable 13). However, the risk of baseline SI in the 25(OH)D insufficiency group was lower than that in the 25(OH)D deficiency group among women (OR [95% CI], 0.59 [0.40–0.87]; *P* = .008) (eTable 14). The 25(OH)D insufficiency group had a higher risk of 1-year incident SI than the deficiency group (OR [95% CI] = 1.78 [1.03–3.09], *P* = .038) in women (eTable 14). Additionally, sex-stratified MR analysis did not reveal a linear or a non-linear relationship between genetically predicted 25(OH)D concentration and the risk of baseline and incident suicidality outcomes in both men (eTable 15) and women (eTable 16).

## Discussion

This study aimed to use traditional observational and MR analyses to investigate the causal effects of 25(OH)D on suicidality in a Chinese early adolescent cohort. Traditional observational analysis did not reveal a significant linear or non-linear association between serum vitamin D concentration and baseline suicidality in the total sample. Interestingly, analysis with the prespecified cut-off values for vitamin D levels revealed that the risk of baseline SI in the serum 25(OH)D deficiency group was significantly higher than that in the 25(OH)D insufficiency group but was not significantly different with that in the 25(OH)D sufficiency group. As different vitamin D cut-off values are used, comparative analysis of findings across different studies is challenging. A cross-sectional study using vitamin D cut-off values similar to those used in this study reported no significant association between vitamin D levels and SI (Park *et al.*, 2016). However, another cross-sectional study classified participants with vitamin D levels < 10, 10–20 and ≥20 ng/mL into deficiency, insufficiency and sufficiency groups, respectively (Kim *et al.*, 2020). The risk of SI in the vitamin D deficiency group was higher than that in the vitamin D sufficiency group (adjusted OR = 1.138; 95% CI = 1.027–1.262). Surprisingly, the risks of 1- and 2-year incident SIs in the 25(OH)D insufficiency group were slightly higher than those in the 25(OH)D deficiency group. However, the association between 25(OH)D levels and the risk of 2-year incident SI disappeared in the sex-stratified analysis, suggesting that this significant association is spurious. Future studies with large sample sizes are needed to confirm the results of incident suicidality analysis with repeated measurements of 25(OH)D. These findings highlighted the importance of sex-stratified analysis rather than controlling for sex as a covariate in statistical models.

In sex-stratified traditional observational analysis, the risk of baseline SI in the 25(OH)D deficiency group was significantly higher than that in the 25(OH)D insufficiency group but was not higher than that in the 25(OH)D sufficiency group in women, indicating the presence of a non-linear association. Indeed, the vitamin D concentration exhibited a significant non-linear association with the risk of baseline SI. Specifically, the risk of baseline SI decreased with increasing 25(OH)D concentration when 25(OH)D was <20 ng/mL and remained stable when 25(OH)D was 20–30 ng/mL. These significant observational associations may be biologically plausible. Vitamin D deficiency can promote inflammation and oxidative stress, increasing the risk of baseline SI. A previous study reported that the concentrations of several biomarkers of inflammation and oxidative stress (malondialdehyde, myeloperoxidase, 3-nitrotyrosine, interleukin-6 and soluble vascular cellular adhesion molecule-1) in participants with vitamin D deficiency were higher than those in participants without deficiency (Filgueiras *et al.*, 2020). Inflammation and oxidative stress are reported to be putative factors involved in the pathophysiology of suicidality (Brundin *et al.*, 2017; Koweszko *et al.*, 2020). Increased inflammation levels have also been identified as a risk factor for various psychiatric disorders, such as bipolar disorder, borderline personality disorder and psychosis (Fraguas *et al.*, 2019; Saccaro *et al.*, 2021), which can partly explain the observational association between vitamin D deficiency and the risk of SI. Additionally, the non-linear association between vitamin D and baseline SI can be partly explained by a threshold effect of vitamin D on inflammation. A recent study reported that vitamin D exerted L-shaped effects on C-reactive protein (CRP) concentration (Zhou and Hypponen, 2023). The CRP level dropped down with increasing vitamin D concentration at low vitamin D concentrations and levelled off when the vitamin D concentration reached 50 nmol/L (Zhou and Hypponen, 2023). Furthermore, at 25(OH)D concentrations >30 ng/mL, the risk of baseline SI increased with an increase in vitamin D concentration. As this study involved a small sample size, the findings of the 25(OH)D sufficiency group should be further confirmed.

However, serum 25(OH)D was not significantly associated with any suicidality outcome in men. These results suggest a sex difference in the association between 25(OH)D and baseline SI. Similarly, a previous study reported that vitamin D concentration was inversely associated with mental health and psychosocial stress only among women (Chen *et al.*, 2020). A plausible explanation for the sex differences is sex-related hormones. In women, low vitamin D levels are associated with decreased estradiol levels (Zhao *et al.*, 2017). Estrogen downregulation can increase the risk of mood disturbances through the activation of neuroinflammation in the hippocampus (Xu *et al.*, 2016) and the upregulation of serotonin neurotransmission (Hernandez-Hernandez *et al.*, 2019). Sex differences in the association between vitamin D and suicidality warrant further investigation.

According to the Bradford Hill criteria, biological plausibility is not a specific criterion for causal inference (Fedak *et al.*, 2015). The presence or absence of biological plausibility did not influence the validity of traditional observational analysis, which is usually determined based on methodological considerations (Savitz, 2021). MR can overcome methodological limitations commonly occurred in traditional observational analysis, such as potential confounders and reverse causality. In this study, MR analysis did not reveal a linear or a non-linear association between genetically predicted 25(OH)D and the risk of suicidality, indicating that vitamin D did not exert causal effects on adolescent suicidality. Similar results were obtained in sex-stratified MR analysis. Revez *et al.* (2020) reported that 25(OH)D concentration did not exert causal effects on various neuropsychiatric traits, including schizophrenia, bipolar disorder, autism spectrum disorder and ADHD. Mulugeta *et al.* (2020) demonstrated that a higher 25(OH)D concentration significantly decreased the risk of depression in observational analysis but genetically predicted 25(OH)D was not associated with depression in MR analysis. A possible explanation for these findings is that the significant association between vitamin D and suicidality observed in previous observational studies is driven by potential confounders and/or reverse causality. Individuals with SI may tend to avoid outdoor activities (leading to decreased sunlight exposure and consequently decreased endogenous vitamin D synthesis) and have a poor appetite with decreased dietary vitamin D intake. Moreover, previous bidirectional MR studies reported the causal effect of several psychiatric disorders on vitamin D (Mulugeta *et al.*, 2020; Revez *et al.*, 2020). However, the possibility of type II error accounting for the null findings in this study cannot be excluded. Thus, a small-to-modest effect may not have been detected owing to the low statistical power. MR studies with a large sample size must be performed in the future.

The findings of this study have several implications for future studies. The non-linear cross-sectional association between vitamin D and SI was significant in women but not in men. This indicates that both non-linearity and sex differences need to be considered in future studies investigating the relationship between vitamin D and suicidality. Moreover, the application of the MR method in nutritional psychiatry is novel, which will aid in the identification of causal nutritional exposures in psychiatry. Future MR studies with large sample sizes should be conducted in this field. Additionally, MR analysis in this study revealed no evidence for the causal effect of vitamin D on suicidality. Thus, the observed association between vitamin D and suicidality in observational studies may be attributed to confounders and/or reverse causality. Accordingly, more attention should be paid to focusing on vitamin D as a potential indicator or a consequence than a causal risk factor for adolescent suicidality in future research examining the relationship between vitamin D and suicidality. Alternatively, the true causal effect of vitamin D on suicidality may be lower than that we had enough power to detect. Thus, further studies are needed with a large sample size.

### Strengths and limitations

This study used data from a longitudinal cohort to investigate the relationship between vitamin D and suicidality in early adolescence. Both linear and non-linear effects were considered using flexible statistical methods to compensate for the limitation of statistical methods with linear hypotheses. Currently, the development of MR in the field of psychiatry is at a relatively early stage (Wootton *et al.*, 2022). MR-based psychiatry research has mainly focused on schizophrenia, major depression, autism spectrum disorders and bipolar disorder, with no studies on suicidality (Saccaro *et al.*, 2022). The findings of this study, which examined the causal effect of vitamin D on suicidality using MR, will promote the application of MR in psychiatry, especially in suicidality research.

Several limitations should be considered when interpreting the findings of this study. Considering that the sample size was relatively small for MR analysis, the statistical power may have not been enough to rule out the possibility of vitamin D exerting a small-to-modest causal effect on suicidality. Further MR studies with a large sample size are needed to confirm the results of this study. Additionally, bidirectional MR analysis was not performed to examine the causal effect of suicidality on vitamin D due to the unavailability of genotype data on suicidality-related SNPs in this study. Future bidirectional MR studies are needed. Furthermore, the study participants were from one school, which limits the generalization of the results. Finally, this study evaluated suicidality outcomes based on self-reporting instead of clinical diagnosis, although the application of objective measures to the general population for research is not feasible.

## Conclusions

In the Chinese early adolescent cohort, traditional observational analysis revealed a significant non-linear association between the 25(OH)D concentration and the risk of baseline SI in women. Moreover, the risk of baseline SI in the 25(OH)D deficiency group was higher than that in the 25(OH)D deficiency group in the total sample. This difference remained significant in women but not in men. However, both linear and non-linear MR methods did not support the causal effect of 25(OH)D on baseline and 1- or 2-year incident suicidality risk. The findings of this study improved our knowledge by indicating that the observed association between vitamin D and suicidality is likely to be driven by potential confounders and/or reverse causation. These findings should be confirmed in a large cohort with sufficient statistical power in the future.

## Data Availability

Data will be available upon reasonable request.

## Supplementary material

For supplementary material accompanying this paper visit http://doi.org/10.1017/S2045796023000665. click here to view supplementary material
