# Vitamin D and suicidality: a Chinese early adolescent cohort and Mendelian randomization study – ERRATUM

**DOI:** 10.1017/S2045796025100152

**Published:** 2025-07-11

**Authors:** Mengyuan Yuan, Yonghan Li, Junjie Chang, Xueying Zhang, Shaojie Wang, Leilei Cao, Yuan Li, Gengfu Wang, Puyu Su

The original publication hosted the incorrect file containing the online supplementary material. The file containing the online supplementary material has been replaced with the correct version.

The Publisher apologises for the error.

## References

[ref1] Yuan M, Li Y, Chang J, Zhang X, Wang S, Cao L, Li Y, Wang G and Su P (2023) Vitamin D and suicidality: a Chinese early adolescent cohort and Mendelian randomization study. *Epidemiology and Psychiatric Sciences* 32, e52. doi:10.1017/S2045796023000665 PMC10465318 37553982

